# Chagas' Disease: Pregnancy and Congenital Transmission

**DOI:** 10.1155/2014/401864

**Published:** 2014-05-15

**Authors:** Ana María Cevallos, Roberto Hernández

**Affiliations:** Departamento de Biología Molecular y Biotecnología, Instituto de Investigaciones Biomédicas, Universidad Nacional Autónoma de México, Apartado Postal 70-228, 04510 México, DF, Mexico

## Abstract

Chagas disease is a chronic infection that kills approximately 12,000 people a year. Mass migration of chronically infected and asymptomatic persons has caused globalization of Chagas disease and has made nonvectorial infection, including vertical and blood-borne transmission, more of a threat to human communities than vectorial infection. To control transmission, it is essential to test all pregnant women living in endemic countries and all pregnant women having migrated from, or having lived in, endemic countries. All children born to seropositive mothers should be tested not only within the first month of life but also at ~6 months and ~12 months of age. The diagnosis is made by identification of the parasite in blood before the age of 6 months and by identification of the parasite in blood and/or positive serology after 10 months of age. Follow up for a year is essential as a significant proportion of cases are initially negative and are only detected at a later stage. If the condition is diagnosed and treated early, the clinical response is excellent and the majority of cases are cured.

## 1. Introduction

Chagas' disease (CD) is a zoonotic infection caused by the hemoflagellate protozoan parasite* Trypanosoma cruzi*. The infection is transmitted to mammalian hosts by a group of hemipteran insects belonging to the family Reduviidae, subfamily Triatominae. In endemic areas, the main mode of transmission is vectorial, by domestic, peridomestic, or sylvatic triatomines. Infection can be also acquired by blood transfusion, organ transplant, congenital infection, and oral transmission from food contaminated with insect faeces. CD is widely associated with poor rural areas, and it is considered as a neglected tropical disease by World Health Organization (WHO). In the early 1990s, the disease was ranked by the World Bank as the most serious of the parasitic diseases in Latin America, with a socioeconomic impact (measured as DALYs—disability-adjusted life years) considerably greater than that of the combined effects of all other parasitic infections [1]. Endemic countries, with the support of the Pan American Health Organization (PAHO), decided to establish regional programmes for the prevention and control of CD. The first program, the Southern Cone Initiative (INCOSUR), was created in 1991 by Argentina, Bolivia, Brazil, Chile, Paraguay, and Uruguay. Later on, the Central American Initiative (IPCA, 1997), the Andean Countries' Initiative (IPA, 1998), the Initiative of the Amazon Countries (AMCHA, 2003), and the Mexican Initiative (Iniciativa para la Vigilancia y el Control de la Enfermedad de Chagas en la República Mexicana, 2004) were created [2]. The main objectives of these initiatives were the control of the vector and the prevention of the transmission of infection by blood transfusion [3]. These multinational initiatives have led to substantial reductions in transmission by* Triatoma infestans*, the principal vector in the Southern Cone countries (Argentina, Brazil, Chile, Paraguay, Bolivia, and Uruguay), and by* Rhodnius prolixus* in Central America. In addition, the risk of transmission by blood transfusion has been substantially reduced throughout Latin America [1]. Estimated annual deaths globally decreased from 45,000 in 1990 to around 11,000 in 2008. The estimated number of infections decreased from 30 million in 1990 to 8 million in 2006 and the annual incidence during this 16-year period fell from 700 000 to 56 000. The burden of CD has been reduced from 2.8 million disability-adjusted life years to less than 500 000 [2].

However, mass migration of chronically infected and asymptomatic persons has caused globalization of CD and it has now been reported in 19 nonendemic areas including the United States, Canada, Europe, Japan, and Australia. In 2010, the World Health Assembly Resolution 63.20 on “Chagas' disease: control and elimination” urged Member States to reduce the burden of CD in nonendemic countries [4]. The resolution also called upon the Director-General to consider an initiative for the prevention and control of CD in nonendemic regions. With this purpose, a group of experts on CD from those European countries, where* T. cruzi*-positive cases had been detected, joined efforts to estimate the prevalence of CD in this region [5]. Their research revealed that, by 2009, 4,290 cases had been diagnosed in Europe, compared with an estimated 68,000 to 122,000 expected cases with an index of underdiagnosis between 94% and 96%. In the Western Pacific Region, an informal consultation held in Nagasaki, Japan, estimated that there were over 1500 infected individuals in Australia and over 3000 in Japan, recognizing the need for local surveillance programmes [6].

In 2004, PAHO focused its attention on the congenital transmission of CD and organized a specific consultation [7]. The advisory group emphasised that, in those regions where achievements or advancements had been made in controlling vectorial and transfusional* T. cruzi* transmission, congenital transmission constituted the main and most persistent form of the parasitosis among the human population. It also recommended that CD should be incorporated to the perinatal information system of the Latin American Centre for Perinatology/Women's and Reproductive Health (CLAP/SMR). CD is now part of the standardized electronic format for the perinatal medical history of CLAP/SMR [8]. PAHO also emphasized the need to consider congenital* T. cruzi* infection as a public-health problem and recommended that each endemic country should elaborate a protocol directed to the prompt detection and specific treatment of detected cases according to the capabilities of the local health services and their epidemiological situation. Vector control programs and serological screening of blood donors are the most effective ways for prevention of congenital infection [9, 10].

The number of cases of congenital CD has been estimated at 14,385 per year in Latin America, at 66–638 per year in the United States, and about 20 to 183 per year in Europe [5, 11, 12]. A systematic review of the literature estimated that in pregnant women with antibodies to* T. cruzi* the global rate of congenital transmission was 4.7% and that countries where the parasite is endemic had a higher rate of congenital transmission compared with countries where it is not endemic (5.0% versus 2.7%) [13]. This difference in prevalence probably reflects the diverse pool of immigrants that the nonendemic countries have, as it has been found that the prevalence of* T. cruzi* infection among immigrant populations normally mirrors the prevalence of the parasite in their countries and regions of origin [14].

## 2. Fertility and Outcome of Pregnancy

Very little is known about the effects of CD in human fertility. In a longitudinal study of the impact of CD in Chile in the 80s, no difference in fertility was identified between seropositive and seronegative women [15]. In animal studies, the majority of* T. cruzi *strains studied had no effect in fertility; however, experimental infection with certain strains has been associated with marked reductions in fertility [16–19]. Further studies are needed to define the overall risk of infertility in CD and its relationship with specific* T. cruzi* strains or lineages.

Evidence for an overall increased risk of abortion or prematurity in seropositive women is inconclusive [20]. However, several studies suggest that maternal chronic infection has no effect on the outcome of pregnancy or on the health of newborns as long as there is no maternal transmission of parasites to the unborn child [21–23]. These studies demonstrate that, when the child is infected, there is an increased risk of premature delivery, low birth weight, and more premature ruptures of the amniotic membranes, effects that may be related to inflammation of the placenta seen in these cases [24–26]. An increased risk of polyhydramnios has also been reported [27].

## 3. Risk Factors for Congenital Transmission

The risk factors for CD congenital transmission are as follows:

 mothers living or migrating from endemic areas,
 mothers living or migrating from areas with high rate of transmission,
 precedent of siblings with congenital infection,
 mother with detectable parasitemias,
 mothers with decreased T-cell-mediated responses to* T. cruzi,*
 coinfection with HIV or Malaria.

The* T. cruzi* infection prevalence among pregnant women varies between different countries, distinct geographical areas, and rural and urban localities from <1% to 70.5% [14]. In recent studies the prevalence rates rose with the increasing maternal age, particularly within those older than 20 years of age, reflecting the success of vector control programmes [28]. However in some rural areas of Bolivia prevalence remains as high as 70.5% and it is expected that the risk of infection will remain elevated in these areas [28–31]. The rate of transmission in endemic countries shows important geographic differences that range between 0% and 18.2% [13, 14].

Congenital transmission of CD may occur during any phase of maternal disease. During the first trimester of pregnancy (weeks 1–12), transmission is probably rare, since the placental intervillous space is not open due to endovascular trophoblast plugging of the spiral arteries. Maternal blood supply becomes continuous and diffuse in the entire placenta only after the 12th week of gestation. Therefore, transmission of blood parasites probably occurs most frequently during the second and third trimesters of pregnancy (prenatal transmission) and perhaps also closer to delivery and during labor (perinatal transmission) through placental breaches/tears [32, 33]. However, the stage of pregnancy when the risk of infection is greatest has not been fully examined as this would entail the systematic evaluation of all pregnancies (including abortions and still births). Furthermore, as most pregnant women acquired the infection prior to conception it is impossible to accurately determine the exact moment of parasite transmission. In studies in which all pregnancies were evaluated, the risk of transplacental transmission was greatest below 34 weeks of gestation, occurring mainly between weeks 22 and 26 [34, 35]. When abortions or stillbirths were not included, congenital transmission occurred predominantly in newborns with a gestational age of 26 to 37 weeks [36]. Three cases of acute infection during pregnancy have been published in detail [37]; in these cases two of the children had no congenital disease and their mothers acquired the disease at weeks 28 and 32 of gestation; the third child was infected and the mother acquired the infection at week 20 of gestation. In another study, two of four women in acute phase of the disease transmitted the disease [38].

Factors that have been implicated in determining the risk of transmission include both maternal factors, such as maternal phase of the disease, immunological status, and obstetrical history, and parasite factors such as the* T. cruzi* strain or the parasitic load.

Infected mothers may transmit the parasite, in one, some, or all their gestations, and may also infect some or all of the siblings in multiple deliveries [21]. Not surprisingly, the clustering of cases within families has been reported [23]. The reasons why some mothers transmit the infection to their offspring and others do not or why one mother can transmit the infection in one pregnancy while not in other pregnancies are not known [14].

Parasitemias may recur with reactivation of chronic disease usually associated with immunosuppression [39]. Pregnancy is known to induce a transient depression of maternal cell-mediated immunity, to prevent rejection of the fetus. An increase in the levels of* T. cruzi* specific IgM has been found in chronically infected pregnant women, suggesting recrudescence of the disease as this antibody is usually found only in the acute phase of the disease [20]. It has been postulated that activation of innate immune defences in pregnant women might contribute to the limitation of the occurrence and severity of congenital infection. Mothers that gave birth to healthy offspring produced higher levels of IL1*β*, IL6, and TNF*α* under stimulation with* T. cruzi* or LPS/PHA than uninfected control mothers and this maternal cellular activation upregulated the capacity of their uninfected neonates to produce such cytokines [40]. Differences in immune responses between transmitting and nontransmitting mothers have been identified. Chronically infected nonpregnant women have increased levels of circulating TNF-*α* and these levels remain increased during pregnancy in women that did not transmit the disease [41]. In contrast, pregnant women that transmitted the parasite had a downregulation of the TNF response. Also, the spontaneous release of TNF by peripheral blood leukocytes was higher in nontransmitting* T. cruzi*-infected pregnant women [42]. As their mothers, noninfected neonates had higher circulating levels of TNF than congenitally infected children. The circulating levels of the soluble receptor TNF-R1 (a TNF regulator) were increased in nontransmitting and transmitting mothers and in infected and noninfected neonates. However, the circulating levels of soluble receptor TNF-R2 were ∼60% higher in infected than in noninfected neonates [42]. A difference in IFN-*γ* response has also been associated with vertical transmission. Mothers that transmitted the infection had decreased production of IFN-*γ* after activation of blood cells with* T. cruzi* lysate and their CD14-positive monocytes expressed less HLA-DR (involved in antigen presentation) and CD54 (involved in cellular adhesion) than infected pregnant women with healthy offspring [43].

Maternal coinfection with* T. cruzi* and HIV results in increasing frequency and severity of congenital CD. Also coinfection with* Plasmodium vivax* results in increased levels of congenital transmission [32].

A high maternal parasitic load has been proposed as a risk factor for transmission. Parasitemias are high during the acute phase of infection and, therefore, transmission rates are expected to be higher in cases of acute disease acquired during pregnancy. In fact, of fifteen reported cases of acute CD during pregnancy, 8 (53%) transmitted the disease to their offspring [20, 37, 38], when the overall rate of transmission is 5% [13]. In contrast, parasitemias are known to be low and recurrent during the indeterminate or chronic phases of infection. However, there is evidence that parasitemias increase during pregnancy [44, 45]. The reported prevalence of parasitemia in pregnant women varies enormously depending on the diagnostic technique employed and on the number of samples taken [28, 29, 44, 45]. Overall rate of parasitemias at some point during pregnancy when more than one sample was examined was 29% when examination of the buffy coat of blood was used [44] and 60.4% when hemoculture was used [45]. The prevalence rate when a single sample was evaluated was 63% using quantitative real time PCR [28]. The time of pregnancy when parasitemia is highest is controversial. In one transversal study, the proportion of positive hemocultures in pregnant women was higher in the first trimester and decreased in women in later stages of pregnancy [45]. In contrast, in a longitudinal study the prevalence of maternal parasitemia was significantly higher during the third trimester of pregnancy than during the first two trimesters [44]. Nevertheless, a direct correlation between high levels of maternal parasitemia and increased risk of transmission has been reported [28, 43, 46, 47].

The role of different genotypes in the risk of congenital infection is unclear.* T. cruzi* parasites have been classified into six different lineages (TcI to TcVI; reviewed in [48]), all of which, with the exception of TcIV, have been identified in human cases of congenital CD [19]. The prevalence of specific* T. cruzi* lineages in cases of congenital disease probably reflects the prevalence of the lineages of the endemic area where they were born [46, 49–51]. The presence of mixed infections is known to occur and therefore the risk of transmitting more than one parasite lineage exists and, in fact, has been reported [49, 52, 53]. As the mixed lineages identified in the newborn are the same as those found in the mother, it is likely that the different* T. cruzi *lineages have a similar potential of crossing the placental barrier [53]. However, animal models have proved that different strains may have different rates of placental invasion and of congenital transmission [17, 54], supporting the idea that the specific genotype of the strain involved is important.

Not surprisingly, factors that determine increased prevalence of chronic Chagas infection such as living in a rural environment, low education, poverty, and poor quality of housing are also factors for increased risk of congenital transmission [26–31].

## 4. Physiopathology of Congenital Infection

Parasites appear to reach the fetus mainly via the hematogenous route across the placenta or through the marginal sinus of the placenta [32, 33]. Less frequently, congenital *T. cruzi* transmission can also occur via the oral route through ingestion of infected amniotic fluid or via the hematogenous route through placental breaches and tears that may occur during delivery [32, 33].

There is evidence that placental innate immune responses can be activated when exposed to* T. cruzi* and that the activation of these responses might reduce or prevent maternal-fetal transmission of the parasites [32, 33]. However, excessive levels of inflammation can be deleterious rather than protective [32, 33].

In cases of aborted infected fetuses, all the placentas showed intense and extensive inflammatory infiltrate along with presence of the parasite; the fetuses also displayed inflammatory infiltrates in all organs studied, demonstrating the presence of catastrophic infection [55, 56]. In one case of maternal acute CD, where the placenta was examined, granulomatous changes, inflammatory infiltrates, and focal necrosis in the chorionic villi were observed. The fibrinoid layer was thicker in some modified villi in which syncytial modifications such as edema and calcification foci were present. Vascular thromboses were also seen. Interestingly, the mother did not transmit the disease [37]. Histopathological differences have been observed between placentas from children born with congenital disease compared to placentas from uninfected children born to seropositive mothers. Chorionitis, chorioamnionitis, and cord edema with lymphocyte infiltration were present in placentas of infected children, whereas such lesions were infiltrated only with polymorphonuclear cells in placentas of noninfected children. Parasites were found in the placentas of infected children, the fibroblasts and macrophages of chorion, membranes, and chorionic plate, mainly in the area of membrane insertion, as well as in cells of Wharton jelly and myocytes of umbilical cord vessels [57]. The authors propose that these results suggest that the maternofetal transmission of parasites occurs mainly through the marginal sinus, spreading into the chorionic plate infecting fibroblasts and macrophages until they reach a fetal vessel, inducing a fetal infection by hematogenous route.

## 5. Clinical Manifestations of Congenital Chagas' Disease

The severity of disease varies enormously from asymptomatic cases to fatal infection and it is related to the level of parasitemia at birth [58]. The reported prevalence of asymptomatic congenital infection varies from 40% to 100% [22, 59–61]. Clinical manifestations can be present at birth or appear within days or weeks after birth [32]. If left untreated, children enter the indeterminate phase of disease with some of them developing chronic disease with typical gastrointestinal and cardiac manifestations [62]. Occasionally, these late symptoms are the first indication of the disease [63].

Many of the signs and symptoms observed in newborns with congenital CD are not specific and may occur with other congenital infections such as toxoplasma or cytomegalovirus (TORCH syndrome). Congenitally* T. cruzi-*infected newborns are frequently premature, have a low birth weight for their gestational age, and have growth retardation, and their APGAR scores are lower than noninfected children [21, 22, 36, 59, 61]. Respiratory distress syndrome is frequently present and can be related to either immaturity of pulmonary function in premature babies and/or pneumonitis associated with parasitism of the alveolar wall [14, 21, 22]. Hepatomegaly, splenomegaly, and jaundice are also common [14, 22, 32, 59, 61].

In severe cases, one or more organs can be affected, most commonly the brain (meningoencephalitis that may be associated with microencephaly) and/or heart (acute myocarditis with cardiomegaly and arrhythmias) [21, 32, 64]. Purpura and oedema (anasarca/fetal hydrops in severe cases) can also occur [14, 32]. The more frequently haematological alterations are anaemia and thrombocytopenia [14, 32]. In children born to mothers infected with HIV and* T. cruzi*, infections were more severe and frequently fatal [65, 66].

In rare occasions, the digestive tract and the eye may be involved. Megaesophagus and megacolon may occur early in congenital disease and can be present at birth [21]. When the gastrointestinal tract is involved, disease is severe and has a high mortality rate [67, 68]. Ocular involvement, with chorioretinitis and opacification of the vitreous body, has also been reported [21, 69, 70].

Mortality rates of approximately 5%, mainly due to myocarditis and meningoencephalitis, have been published [14]. Torrico et al. [22] described mortality rates of up to 13% in a cohort of infected infants studied between 1992 and 1994, while the mortality rate dropped to 2% 6 years later, probably as a result of the improvement of the socioeconomic environment in Bolivia. Mortality was higher in infected children born prematurely with severe clinical manifestations.

## 6. Diagnosis

Symptomatic congenital CD should be considered in any newborn with clinical findings suggestive of a vertically transmitted infection such as toxoplasma or rubella whose mother has positive serology for* T. cruzi* or has a sibling with CD.

 Criteria for suspicion of symptomatic congenital Chagas' disease are as follows:
   signs and symptoms of vertically transmitted infection:
     prematurity,
     small for gestational age,
     low APGAR score,
     respiratory distress syndrome,
     hepato-/splenomegaly,
     jaundice;
   
*T. cruzi *seropositive mother (by 2 different standard tests);
   sibling with congenital Chagas' disease;
   evidence of myocarditis or meningoencephalitis.

Increased awareness of the possibility of congenital transmission is essential as failure to test for and treat this infection may lead to the death of the infant [71].

Congenital infection is frequently asymptomatic; therefore, all children born to seropositive mothers should be tested for CD. Unfortunately, this is not routinely done even in endemic areas. A study in Argentina showed that only 17% of multiparous seropositive pregnant mothers that had been sent to a reference health centre had offspring that had been tested for CD [72]. In endemic areas it has been estimated that for each diagnosed case there is at least 6 undiagnosed cases [73, 74]. In nonendemic areas the rate of underdiagnosis is over 90% [5]. Routine testing of older children born to seropositive mothers can identify some of these cases [75].

According to the Technical Group on “Prevention and Control of Congenital Transmission and Case Management of Congenital Infections” (IVa) of WHO Programme on Control of CD, the gold standard for diagnosis of congenital infection is the detection of blood parasites at any time after birth or a positive* T. cruzi*—specific serology in infants aged >8 months (when previous transmission by vectors and blood transfusion has been ruled out) [76]. In the experience of Instituto Nacional de Parasitología Dr. Mario Fatala Chaben, a referral centre for pregnant seropositive women and for the diagnosis of congenital CD, a positive diagnosis could only be established within the first month of life in 44.6% of the babies, in 24.3% of cases diagnosis was not made until the 5th month, and in 31.1% diagnosis could not be confirmed until 6 to 12 months of age [77]. Therefore, it is essential to follow up children born to seropositive mothers and perform routine testing for both searching for parasite in blood and monitoring their antibody response until the end of the first year of life [73, 78]. It is regrettable that more than 55% of children are lost to followup after the age of 6 months and that less than one in two congenital cases is correctly identified and treated [61, 77–79].

## 7. Identification of the Parasite

Early diagnosis in children born to seropositive mothers depends on the detection of blood parasites, when maternal* T. cruzi *antibodies could still be present. The microscopical observation of fresh blood between slide and coverslip can easily disclose the presence of the parasites because of their motility. Thin- and thick-stained blood smears allow detection of the morphological characteristic of the parasite. When the parasite load is low, a concentration method is required, either by a Strout test (where serum is spun and the resulting pellet is examined) or by examination of the white buffy coat (the leucocyte layer that separates red cells from plasma in a haematocrit test, also known as microhematocrit method) [80, 81]. These methods are particularly useful when there are high levels of parasitemia such as cases of acute infection or during the first months of life in cases of congenital infection. It is worthwhile to test 4 microhematocrit tubes at a time, as this modified procedure increases the sensitivity of the test to detection of parasitemia levels as low as 50 parasites per milliliter [58].

Indirect methods for the identification of the parasite are generally used when the parasitic load is low (chronic disease) and require the expansion of the parasite population under laboratory conditions that are not available everywhere. There are two ways of growing the parasite, by hemoculture or by xenodiagnosis (technique that exposes suspected infected tissue to a vector and then examines the vector for the presence of the particular pathogen). These tests may take several weeks to become positive but are very sensitive [47, 82, 83].

The histopathological analysis of the placenta is not considered a good diagnostic test either because the presence of parasites may be missed or because its presence, although being suggestive, does not necessarily indicate congenital infection. Placentas of uninfected newborns from infected mothers can present with parasites and severe histological changes without being associated with fetal infection [14, 21, 32, 64]. Also placentas of children with congenital infection may not show abnormalities [64].

## 8. Molecular Diagnosis

The identification of parasite antigens or DNA in blood can suggest the presence of infection. The detection of* T. cruzi* soluble antigens in urines and serum by capture ELISA assays has been proposed for diagnosis of congenital cases. However, these tests did not detect all infected cases [32].

The amplification of* T. cruzi* nuclear or kinetoplastic DNA is considered “under evaluation” by the World Health Organization but has been used to detect low levels of parasitemia in congenital cases, and results can be obtained in a short time [76]. However, the presence of parasite DNA in the blood of the newborn does not necessarily indicate active infection as it does not prove that the parasites are viable [14, 32]. It has been suggested that the high rates of congenital infection reported by some authors may be due to the amplification of trace amounts of DNA from the mother and not the presence of live parasites [32]. Therefore, it is recommended that the test should be confirmed with subsequent samples at approximately 3 and 9 months after birth [78, 84]. Although this technique has not been fully validated, it has a good predictive value and has the advantage of not needing a specially trained observer [78].

## 9. Serological Diagnosis

In the first months of life conventional serology is not useful due to the transference of maternal antibodies through the placenta. It has been suggested that a test using shed acute phase antigen (SAPA) only detects acute or congenital infection, as this antigen is not present in the chronic phase of disease [85, 86]. However, antibodies against this antigen were detected in ~80% of patients with intermediate disease [87]. More studies are needed to determine the value of this test.

After the first 9 months maternal antibodies have disappeared and therefore the presence of specific anti-*T. cruzi *antibodies with conventional serological test is considered diagnosis. As with chronic cases the use of two different serological tests is recommended (IHA, IF, or ELISA). In some cases, diagnosis is only made by a positive serology [47, 78]. Failure to detect parasites in these children is probably due to low parasitemias and reflects the need to develop more sensitive assays and the importance of performing a serological test at the age of 12 months [73, 78].

## 10. Antenatal Screening

According to the recommendations of the Technical Group IVa on “Prevention and Control of Congenital Transmission and Case Management of Congenital Infections,” serological testing is recommended for pregnant women (i) who are living in disease-endemic areas, (ii) who are living in disease nonendemic areas and have occasionally received blood transfusion in disease-endemic areas, and (iii) who are living in disease nonendemic areas and are born or have lived previously in disease-endemic areas or whose mothers were born in such areas [76].

This group also recommended that CD should be systematically investigated in siblings and relatives of infected mothers (serological investigation), and positive cases should be clinically evaluated and treated accordingly [76].

## 11. Treatment

Treatment is generally successful and without the adverse reactions seen in adults if administered within the first year of life [76, 88]. All children must be followed up after treatment to ensure that they have eliminated the parasite. The long term prognosis has not been well studied, but in a communication of their 30-year experience, Moya et al. (2005) reported that, for children treated before the age of three, they were cured and that, at 13–15 years of age, they had no evidence of cardiac abnormalities [64]. Failure to diagnose and treat the infection may result in chronic symptomatic disease [63].

Treatment should be given according to the guidelines established by the Technical Group IVa on “Prevention and Control of Congenital Transmission and Case Management of Congenital Infections” [76].

All cases of congenital* T. cruzi* infection should be treated as soon as the diagnosis has been confirmed with either benznidazole or nifurtimox.

The recommended dose of benznidazole in infants, as in adults, is 5–7 mg/kg per day; doses of benznidazole up to 10 mg/kg per day can be used in neonates and infants aged <1 year. Benznidazole is manufactured by Laboratório Farmacêutico do Estado de Pernambuco (LAPEFE, Brazil) and is available in tablets of 100 mg through “Masters” (Davie, Florida, United States; Elstree, Hertfordshire, United Kingdom), the WHO, and the PAHO. This technical group suggests that dispersal tablets of 12.5 mg should become available to facilitate the preparation of paediatric suspensions.

The recommended doses of nifurtimox in neonates and infants are 10–15 mg/kg per day. Nifurtimox is manufactured by Bayer and is available in tablets of 120 mg through WHO and PAHO.

Treatment with either drug, should be administered orally in one dose in low-weight neonates or, preferably, in divided doses of two to three subdoses; precautions should to be taken to obtain appropriate dosage of active drug, since the currently available tablets have to be crushed and used as a suspension.

The recommended duration of treatment is 60 days and should not be <30 days.

## 12. Chagas' Disease and Breastfeeding

CD can be acquired through the ingestion of contaminated food or water. Therefore, the possibility of transmission through breastfeeding may be particularly relevant, particularly because such transmission could be preventable. The risk of transmission through this route has been recently reviewed [89]. In mice, oral transmission of* T. cruzi* infection through human milk contaminated with trypomastigotes is possible, although natural transmission through breastfeeding has not been clearly demonstrated. In humans, contamination of milk with trypomastigotes has been described; however, except for some dated and inconclusive cases, transmission through breastfeeding has not been reported [89]. More studies are needed to fully evaluate the risk of infection through breastfeeding.

Exclusive breastfeeding is an ideal way to provide nutrition during the first 6 months of life, and interruption of breastfeeding in resource-poor settings does not seem feasible unless the risks clearly outweigh possible benefits. Therefore, the discontinuation of breastfeeding by mothers with chronic CD is not recommended [89, 90]. However, breastfeeding by mothers, with acute CD or with fissures and bleeding nipples, should be avoided.

## 13. Concluding Remarks

Congenital CD may occur in any part of the world and the lack of well-established surveillance programs means that the diagnosis is largely missed. Identification of all affected children involves testing all pregnant women at risk of infection, that is, either living in an endemic area or having migrated from an endemic area. All children born to seropositive mothers should be tested not only within the first month of life but also at ~6 months and ~12 months of age. The diagnosis is made by identification of the parasite using standardized micromethods before 6 months and by a positive serology after 10 months of age. Followup for a year is essential as a significant proportion of cases are initially negative and are only detected at a later stage by either detection of blood parasites or by seroconversion. The success of the followup depends on establishing good followup routines in primary care settings and on extensive counselling of the mothers emphasizing the relevance of control even in asymptomatic and apparently healthy children [77]. Early diagnosis is important because, within the first year of life, the response to treatment is almost 100% and well tolerated. Siblings of children with congenital infection should also be studied.

In their first report on neglected diseases the WHO recognized that the movement of CD to areas previously considered nonendemic, resulting from increasing population mobility between Latin America and the rest of the world, represents a serious public-health challenge (report of neglected diseases) [91]. It also expressed a preoccupation that, in places where health professionals have little knowledge or experience of the disease and its control, the diagnosis of CD will be missed and left untreated. Therefore, it is important that developed country researchers establish bilateral and multilateral CD collaborations to help health care professionals in their regions to learn from the decades of experience of Latin American scientists and to provide the resources and a collaborative platform to advance the search for better ways to diagnose, treat, and prevent the disease [92].

## References

[1] Dias JC, Silveira AC, Schofield CJ (2002). The impact of Chagas disease control in Latin America: a review. *Memorias do Instituto Oswaldo Cruz*.

[2] Pan American Health Organization (2014). Chagas' disease: factsheet. *General Information and Distribution*.

[3] Pan American Health Organization Chagas' disease: Scientific and Technical Materials.

[4] World Health Organization (2010). Chagas disease: control and elimination. *The Sixty-Third World Health Assembly*.

[5] Basile L, Jansa JM, Carlier Y (2011). Chagas disease in European countries: the challenge of a surveillance system. *Euro Surveillance*.

[6] World Health Organization (2011). Informal consultation on Chagas' disease in the Western Pacific region. *WHO Western Pacific*.

[7] Pan American Health Organization PAHO Consultation on Congenital Chagas Disease, Its Epidemiology and Management.

[8] Mainero L, Martínez G, Rubino M, de Mucio B, Díaz Rossello JL, Fescina RH (2010). *Sistema Informático Perinatal (SIP): Manual de Uso del Programa Para el Análisis y Aprovechamiento de la Información*.

[9] Carlier Y, Torrico F (2003). Congenital infection with *Trypanosoma cruzi*: from mechanisms of transmission to strategies for diagnosis and control. *Revista da Sociedade Brasileira de Medicina Tropical*.

[10] Pan American Health Organization Regional Consultation on Organization and Structure of Health Care for the sick or infected with *Trypanosoma cruzi* (Chagas' disease).

[11] Pan American Health Organization (2006). Estimación cuantitativa de la enfermedad de Chagas en las Américas. *Organización Panamericana de la Salud*.

[12] Yadon ZE, Schmunis GA (2009). Congenital Chagas disease: estimating the potential risk in the United States. *The American Journal of Tropical Medicine and Hygiene*.

[13] Howard E, Xiong X, Carlier Y, Sosa-Estani S, Buekens P (2014). Frequency of the congenital transmission of *Trypanosoma cruzi*: a systematic review and meta-analysis. *BJOG*.

[14] Oliveira I, Torrico F, Muoz J, Gascon J (2010). Congenital transmission of Chagas disease: a clinical approach. *Expert Review of Anti-Infective Therapy*.

[15] Schenone H, del Contreras CM, Borgono JM (1985). Congenital Chagas' disease in Chile. Longitudinal study of the reproductivity of women with or without Chagas' disease and of some parasitological and clinical parameters of them and their corresponding children. *Boletín Chileno de Parasitología*.

[16] Cappa SMG, Mirkin GA, Solana ME, Tekiel VS (1999). *Trypanosoma cruzi* pathology. Strain dependent?. *Medicina*.

[17] Solana ME, Celentano AM, Tekiel V, Jones M, González Cappa SM (2002). *Trypanosoma cruzi*: effect of parasite subpopulation on murine pregnancy outcome. *The Journal of Parasitology*.

[18] Alkmim-Oliveira SM, Costa-Martins AG, Kappel HB, Correia D, Ramirez LE, Lages-Silva E (2013). *Trypanosoma cruzi* experimental congenital transmission associated with TcV and TcI subpatent maternal parasitemia. *Parasitology Research*.

[19] Cencig S, Coltel N, Truyens C, Carlier Y (2013). Fertility, gestation outcome and parasite congenital transmissibility in mice infected with TcI, TcII and TcVI genotypes of *Trypanosoma cruzi*. *PLoS Neglected Tropical Diseases*.

[20] Brabin L (1992). The epidemiological significance of Chagas’ disease in women. *Memorias do Instituto Oswaldo Cruz*.

[21] Bittencourt AL (1992). Possible risk factors for vertical transmission of Chagas’ disease. *Revista do Instituto de Medicina Tropical de Sao Paulo*.

[22] Torrico F, Alonso-Vega C, Suarez E (2004). Maternal *Trypanosoma cruzi* infection, pregnancy outcome, morbidity, and mortality of congenitally infected and non-infected newborns in Bolivia. *The American Journal of Tropical Medicine and Hygiene*.

[23] Negrette OS, Mora MC, Basombrío MA (2005). High prevalence of congenital *Trypanosoma cruzi* infection and family clustering in Salta, Argentina. *Pediatrics*.

[24] Duaso J, Rojo G, Cabrera G (2010). *Trypanosoma cruzi* induces tissue disorganization and destruction of chorionic villi in an *ex vivo* infection model of human placenta. *Placenta*.

[25] Duaso J, Yanez E, Castillo C (2012). Reorganization of extracellular matrix in placentas from women with asymptomatic Chagas disease: mechanism of parasite invasion or local placental defense?. *Journal of Tropical Medicine*.

[26] Apt W, Zulantay I, Arnello M (2013). Congenital infection by *Trypanosoma cruzi* in an endemic area of Chile: a multidisciplinary study. *Transactions of the Royal Society of Tropical Medicine and Hygiene*.

[27] Hernandez-Matheson IM, Frankowski RF, Held B (1983). Foeto-maternal morbidity in the presence of antibodies to *Trypanosoma cruzi*. *Transactions of the Royal Society of Tropical Medicine and Hygiene*.

[28] Bern C, Verastegui M, Gilman RH (2009). Congenital *Trypanosoma cruzi* Transmission in Santa Cruz, Bolivia. *Clinical Infectious Diseases*.

[29] Brutus L, Schneider D, Postigo J, Romero M, Santalla J, Chippaux JP (2008). Congenital Chagas disease: diagnostic and clinical aspects in an area without vectorial transmission, Bermejo, Bolivia. *Acta Tropica*.

[30] Chippaux J-P, Santalla JA, Postigo JR (2009). Sensitivity and specificity of Chagas Stat-Pak test in Bolivia. *Tropical Medicine & International Health*.

[31] Cucunubá ZM, Flórez AC, Cárdenas A (2012). Prevalence and risk factors for Chagas disease in pregnant women in Casanare, Colombia. *The American Journal of Tropical Medicine and Hygiene*.

[32] Carlier Y, Truyens C, Telleria J, Tibayrenc M (2010). Maternal-fetal transmission of *Trypanosoma cruzi*. *American Trypanosomiasis—Chagas Disease One Hundred Years of Research*.

[33] Carlier Y, Truyens C, Deloron P, Peyron F (2012). Congenital parasitic infections: a review. *Acta Tropica*.

[34] Bittencourt AL, Barbosa HS (1972). Incidence of congenital transmission of Chagas’ disease in abortion. *Revista do Instituto de Medicina Tropical de Sao Paulo*.

[35] Bittencourt AL, Barbosa HS, Santos I, Ramos ME (1974). Incidence of congenital transmission of Chagas' disease in term pregnancies. *Revista do Instituto de Medicina Tropical de Sao Paulo*.

[36] Azogue E, la Fuente C, Darras C (1985). Congenital Chagas’ disease in Bolivia: epidemiological aspects and pathological findings. *Transactions of the Royal Society of Tropical Medicine and Hygiene*.

[37] Moretti E, Basso B, Castro I (2005). Chagas’ disease: study of congenital transmission in cases of acute maternal infection. *Revista da Sociedade Brasileira de Medicina Tropical*.

[38] Salas NA, Cot M, Schneider D (2007). Risk factors and consequences of congenital Chagas disease in Yacuiba, south Bolivia. *Tropical Medicine & International Health*.

[39] Pinazo MJ, Espinosa G, Cortes-Lletget C (2013). Immunosuppression and Chagas disease: a management challenge. *PLoS Neglected Tropical Diseases*.

[40] Vekemans J, Truyens C, Torrico F (2000). Maternal *Trypanosoma cruzi* infection upregulates capacity of uninfected neonate cells to produce pro- and anti-inflammatory cytokines. *Infection and Immunity*.

[41] Cardoni RL, García MM, de Rissio AM (2004). Proinflammatory and anti-inflammatory cytokines in pregnant women chronically infected with *Trypanosoma cruzi*. *Acta Tropica*.

[42] García MM, de Rissio AM, Villalonga X, Mengoni E, Cardoni RL (2008). Soluble tumor necrosis factor (TNF) receptors (sTNF-R1 and -R2) in pregnant women chronically infected with *Trypanosoma cruzi* and their children. *The American Journal of Tropical Medicine and Hygiene*.

[43] Hermann E, Truyens C, Alonso-Vega C (2004). Congenital transmission of *Trypanosoma cruzi* is associated with maternal enhanced parasitemia and decreased production of interferon-*γ* in response to parasite antigens. *The Journal of Infectious Diseases*.

[44] Brutus L, Castillo H, Bernal C (2010). Detectable *Trypanosoma cruzi* parasitemia during pregnancy and delivery as a risk factor for congenital Chagas disease. *The American Journal of Tropical Medicine and Hygiene*.

[45] Siriano LDR, Luquetti AO, Avelar JB, Marra NL, de Castro AM (2011). Chagas disease: increased parasitemia during pregnancy detected by hemoculture. *The American Journal of Tropical Medicine and Hygiene*.

[46] Virreira M, Truyens C, Alonso-Vega C (2007). Comparison of *Trypanosoma cruzi* lineages and levels of parasitic DNA in infected mothers and their newborns. *The American Journal of Tropical Medicine and Hygiene*.

[47] Bua J, Volta BJ, Velazquez EB, Ruiz AM, Rissio AM, Cardoni RL (2012). Vertical transmission of *Trypanosoma cruzi* infection: quantification of parasite burden in mothers and their children by parasite DNA amplification. *Transactions of the Royal Society of Tropical Medicine and Hygiene*.

[48] Zingales B, Miles MA, Campbell DA (2012). The revised *Trypanosoma cruzi* subspecific nomenclature: rationale, epidemiological relevance and research applications. *Infection, Genetics and Evolution*.

[49] Burgos JM, Altcheh J, Bisio M (2007). Direct molecular profiling of minicircle signatures and lineages of *Trypanosoma cruzi* bloodstream populations causing congenital Chagas disease. *International Journal for Parasitology*.

[50] Corrales RM, Mora MC, Negrette OS (2009). Congenital Chagas disease involves *Trypanosoma cruzi* sub-lineage IId in the northwestern province of Salta, Argentina. *Infection, Genetics and Evolution*.

[51] Virreira M, Alonso-Vega C, Solano M (2006). Congenital Chagas disease in Bolivia is not associated with DNA polymorphism of *Trypanosoma cruzi*. *The American Journal of Tropical Medicine and Hygiene*.

[52] Ortiz S, Zulantay I, Solari A (2012). Presence of *Trypanosoma cruzi* in pregnant women and typing of lineages in congenital cases. *Acta Tropica*.

[53] Garcia A, Ortiz S, Iribarren C, Bahamonde MI, Solari A (2014). Congenital co-infection with different *Trypanosoma cruzi* lineages. *Parasitology International*.

[54] Andrade SG (1982). The influence of the strain of *Trypanosoma cruzi* in placental infections in mice. *Transactions of the Royal Society of Tropical Medicine and Hygiene*.

[55] Bittencourt AL, Barbosa HS (1972). Importance of the study of the macerated fetus for the diagnosis of congenital Chagas’ disease. *Revista do Instituto de Medicina Tropical de Sao Paulo*.

[56] Altemani AM, Bittencourt AL, Lana AM (2000). Immunohistochemical characterization of the inflammatory infiltrate in placental Chagas’ disease: a qualitative and quantitative analysis. *The American Journal of Tropical Medicine and Hygiene*.

[57] Fernandez-Aguilar S, Lambot MA, Torrico F (2005). Placental lesions in human *Trypanosoma cruzi* infection. *Revista da Sociedade Brasileira de Medicina Tropical*.

[58] Torrico MC, Solano M, Guzmán JM (2005). Estimation of the parasitemia in *Trypanosoma cruzi* human infection: high parasitemias are associated with severe and fatal congenital Chagas disease. *Revista da Sociedade Brasileira de Medicina Tropical*.

[59] Freilij H, Altcheh J (1995). Congenital Chagas’ disease: diagnostic and clinical aspects. *Clinical Infectious Diseases*.

[60] Contreras S, Fernandez MR, Agüero F, Desse Desse J, Orduna T, Martino O (1999). Congenital Chagas-Mazza disease in Salta, Argentina. *Revista da Sociedade Brasileira de Medicina Tropical*.

[61] Blanco SB, Segura EL, Cura EN (2000). Congenital transmission of *Trypanosoma cruzi*: an operational outline for detecting and treating infected infants in north-western Argentina. *Tropical Medicine & International Health*.

[62] Schijman AG, Mushahwar IK (2006). Congenital Chagas disease. *Congenital and Other Related Infectious Diseases of the Newborn*.

[63] Imai K, Maeda T, Sayama Y (2014). Mother-to-child transmission of congenital Chagas disease, Japan. *Emerging Infectious Diseases*.

[64] Moya P, Basso B, Moretti E (2005). Congenital Chagas disease in Córdoba, Argentina: epidemiological, clinical, diagnostic, and therapeutic aspects. Experience of 30 years of follow up. *Revista da Sociedade Brasileira de Medicina Tropical*.

[65] Freilij H, Altcheh J, Muchinik G (1995). Perinatal human immunodeficiency virus infection and congenital Chagas’ disease. *Pediatric Infectious Disease Journal*.

[66] Sartori AM, Ibrahim KY, Westphalen EVN (2007). Manifestations of Chagas disease (American trypanosomiasis) in patients with HIV/AIDS. *Annals of Tropical Medicine and Parasitology*.

[67] Bittencourt AL, Vieira GO, Tavares HC (1984). Esophageal involvement in congenital Chagas’ disease. Report of a case with megaesophagus. *The American Journal of Tropical Medicine and Hygiene*.

[68] Atías A (1994). A case of congenital chagasic megaesophagus: evolution until death caused by esophageal neoplasm, at 27 years of age. *Revista Medica de Chile*.

[69] Atías A, Morales M, Muñoz P, Barría M (1985). Ocular involvement in congenital Chagas’ disease. *Revista Chilena de Pediatria*.

[70] Berberian G, Rosanova MT, Kaldzielski C, Paulin P, Castro G, Galina L (2013). Ocular involvement in congenital Chagas disease. *Archivos Argentinos de Pediatría*.

[71] Flores-Chávez M, Faez Y, Olalla JM (2008). Fatal congenital Chagas' disease in a non-endemic area: a case report. *Cases Journal*.

[72] García MM, de Rissio AM, Ruiz AM (2007). Estudio epidemiológico y diagnóstico de la infección por *Trypanosoma cruzi* en mujeres embarazadas del Centro Nacional de Referencia Instituto Nacional de Parasitología Dr. Mario Fatala Chaben. *Archivos Argentinos de Epidemiología*.

[73] de Rissio AM, Scollo K, Cardoni RL (2009). Maternal fetal-transmission of *Trypanosoma cruzi* in Argentina. *Medicina*.

[74] Gürtler RE, Segura EL, Cohen JE (2003). Congenital transmission of *Trypanosoma cruzi* infection in Argentina. *Emerging Infectious Diseases*.

[75] Rassi A, Neto VA, Rassi GG (2004). A retrospective search for maternal transmission of Chagas infection from patients in the chronic phase. *Revista da Sociedade Brasileira de Medicina Tropical*.

[76] Carlier Y, Torrico F, Sosa-Estani S (2011). Congenital Chagas disease: recommendations for diagnosis, treatment and control of newborns, siblings and pregnant women. *PLoS Neglected Tropical Diseases*.

[77] de Rissio AM, Riarte AR, García MM, Esteva MI, Ruiz AM, Quaglino M (2010). Congenital *Trypanosoma cruzi* infection. Efficacy of its monitoring in an urban reference health center in a non-endemic area of Argentina. *The American Journal of Tropical Medicine and Hygiene*.

[78] Bua J, Volta BJ, Perrone AE (2013). How to improve the early diagnosis *of Trypanosoma cruzi* infection: relationship between validated conventional diagnosis and quantitative DNA amplification in congenitally infected children. *PLoS Neglected Tropical Diseases*.

[79] Russomando G, de Tomassone MMC, de Guillen I (1998). Treatment of congenital Chagas’ disease diagnosed and followed up by the polymerase chain reaction. *The American Journal of Tropical Medicine and Hygiene*.

[80] Strout RG (1962). A method for concentrating hemoflagellates. *The Journal of Parasitology*.

[81] Feilij H, Muller L, Cappa SMG (1983). Direct micromethod for diagnosis of acute and congenital Chagas’ disease. *Journal of Clinical Microbiology*.

[82] Murcia L, Carrilero B, Munoz-Davila MJ, Thomas MC, López MC, Segovia M (2013). Risk factors and primary prevention of congenital Chagas disease in a non endemic country. *Clinical Infectious Diseases*.

[83] Saavedra M, Zulantay I, Apt W, Martínez G, Rojas A, Rodríguez J (2013). Chronic Chagas disease: PCR-xenodiagnosis without previous microscopic observation is a useful tool to detect viable *Trypanosoma cruzi*. *Biological Research*.

[84] Diez CN, Manattini S, Zanuttini JC, Bottasso O, Marcipar I (2008). The value of molecular studies for the diagnosis of congenital Chagas disease in northeastern Argentina. *The American Journal of Tropical Medicine and Hygiene*.

[85] Reyes MB, Lorca M, Muñoz P, Frasch AC (1990). Fetal IgG specificities against *Trypanosoma cruzi* antigens in infected newborns. *Proceedings of the National Academy of Sciences of the United States of America*.

[86] Mallimaci MC, Sosa-Estani S, Russomando G (2010). Early diagnosis of congenital *Trypanosoma cruzi* infection, using shed acute phase antigen, in Ushuaia, Tierra del Fuego, Argentina. *The American Journal of Tropical Medicine and Hygiene*.

[87] Brenière SF, Yaksic N, Telleria J (1997). Immune response to *Trypanosoma cruzi* shed acute phase antigen in children from an endemic area for Chagas' disease in Bolivia. *Memórias do Instituto Oswaldo Cruz*.

[88] Altcheh J, Moscatelli G, Moroni S, Garcia-Bournissen F, Freilij H (2011). Adverse events after the use of benznidazole in infants and children with Chagas disease. *Pediatrics*.

[89] Norman FF, López-Vélez R (2013). Chagas disease and breast-feeding. *Emerging Infectious Diseases*.

[90] Bittencourt AL, Sadigursky M, da Silva AA (1988). Evaluation of Chagas’ disease transmission through breast-feeding. *Memorias do Instituto Oswaldo Cruz*.

[91] World Health Organization (2010). First WHO report on neglected tropical diseases: working to overcome the global impact of neglected tropical diseases.

[92] Gascon J, Bern C, Pinazo MJ (2010). Chagas disease in Spain, the United States and other non-endemic countries. *Acta Tropica*.

